# Association between Maternal Pre-pregnancy Body Mass Index and Breastfeeding Duration in Taiwan: A Population-Based Cohort Study

**DOI:** 10.3390/nu12082361

**Published:** 2020-08-07

**Authors:** Chi-Nien Chen, Hung-Chen Yu, An-Kuo Chou

**Affiliations:** 1 Department of Pediatrics, National Taiwan University Hospital Hsin-Chu Branch, Hsin-Chu 30059, Taiwan; dtped124@gmail.com; 2 School of Nursing, College of Medicine, National Taiwan University, Taipei 10051, Taiwan; 101998sunny@gmail.com; 3 Institute of Health Policy and Management, College of Public Health, National Taiwan University, Taipei 10055, Taiwan

**Keywords:** maternal underweight, maternal obesity, breastfeeding, population cohort, pre-pregnancy body mass index

## Abstract

An association between high pre-pregnancy body mass index (BMI) and early breastfeeding cessation has been previously observed, but studies examining the effect of underweight are still scant and remain inconclusive. This study analyzed data from a nationally representative cohort of 18,312 women (mean age 28.3 years; underweight 20.1%; overweight 8.2%; obesity 1.9%) who delivered singleton live births in 2005 in Taiwan. Comprehensive face-to-face interviews and surveys were completed at 6 and 18 months postpartum. BMI status and breastfeeding duration were calculated from the self-reported data in the questionnaires. In the adjusted ordinal logistic regression model, maternal obesity and underweight had a higher odds of shorter breastfeeding duration compared with normal-weight women. The risk of breastfeeding cessation was significantly higher in underweight women than in normal-weight women after adjustments in the logistic regression model (2 m: aOR = 1.11, 95% CI = 1.03–1.2; 4 m: aOR = 1.32, 95% CI = 1.21–1.43; 6 m: aOR = 1.3, 95% CI = 1.18–1.42). Our findings indicated that maternal underweight and obesity are associated with earlier breastfeeding cessation in Taiwan. Optimizing maternal BMI during the pre-conception period is essential, and future interventions to promote and support breastfeeding in underweight mothers are necessary to improve maternal and child health.

## 1. Introduction

Breastfeeding is a key strategy to improve maternal and child health. The benefits for infants include the reduction of risk of infectious disorders, obesity, allergic disorders, and developmental delays [1,2]. For mothers, breastfeeding could protect against postpartum weight retention, cardiovascular diseases, and malignancy, including breast, ovarian and endometrial cancers [3].

Although the World Health Organization (WHO) and the American Academy of Pediatrics (AAP) have recommended exclusive breastfeeding for the first six months of life [4,5], recent data suggest that less than 37% of women could fulfill this goal worldwide [2]. Factors associated with the low breastfeeding rate are multifaceted. Potential risk factors for breastfeeding cessation are maternal smoking behavior, delivery mode, parity, dyad separation, maternal educational status [6], paternal support [7], maternal perception of lacking sufficient milk supply, mastitis, infants’ failure to thrive [8], predelivery breastfeeding education [9], and maternal obesity [10].

The epidemic of maternal obesity is a major public health concern today [11]. More than 30% of American women of reproductive age are obese [12]. Overweight and obese women may not only have a greater risk of adverse perinatal outcomes [13], but may also have more difficulty in continuing to breastfeed after delivery, due to a decreased prolactin secretion response to suckling [14] and delayed lactogenesis II [15]. However, observational studies have been conducted, to determine the possible associations between pre-pregnancy body mass index (pBMI) and breastfeeding practices, and the evidence remains inconclusive [16,17,18,19,20,21]. Less is known about the effects of maternal pre-pregnancy underweight status on breastfeeding behavior. In one previous study, mothers with pre-pregnancy underweight status had a higher risk of any breastfeeding cessation within two months postpartum [21]. However, Giovannini et al. reported no difference between underweight and normal-weight mothers for the duration of breastfeeding [22]. The evidence of this association in Asian populations is scant, and there is not enough data to show the relationship between pBMI and breastfeeding practices. Only a few studies conducted in Japan and China have reported that pre-pregnancy obesity [17,23] and underweight [21] may have a negative effect on breastfeeding duration.

The inconsistency in previous literature might result from differences in study designs, study populations, sample sizes and various breastfeeding outcome definitions. In our previous study, the prevalence of underweight was greater in Taiwan than in the United States and European countries [11,24]. It is unclear whether pre-pregnancy underweight status may have a certain effect on breastfeeding behaviors from a population with a higher proportion of women who are underweight. Therefore, to address these knowledge gaps and study limitations in previous literature, we conducted a secondary analysis study by using data from a prospective birth cohort in Taiwan, to explore the association between pBMI and breastfeeding practices.

## 2. Materials and Methods

### 2.1. Data Source and Study Population

The study population included in the present analysis was from the Taiwan Birth Cohort Study (TBCS), in which the Taiwan Health Promotion Administration supervised a prospective longitudinal nationally representative birth cohort. Study participants were mother-child dyads born between 1 January 2005 and 31 December 2005. The TBCS enrolled postpartum women by a two-stage, stratified, systematic random sampling method from 369 cities and towns in Taiwan. The first and second face-to-face interviews and questionnaire surveys with mothers or primary caregivers were completed at 6 and 18 months postpartum, between 2005 and 2007. Initially, 24,200 (11.7% sampling rate of all children born in 2005) mother-child dyads were selected, and 21,648 and 20,559 of them completed the first and second surveys, respectively. Participants with multiple pregnancies (twins, triplets or more), those with missing or invalid data (pre-pregnancy body weight < 20 kg or > 200 kg), or mothers with major illness or pre-existing diabetes were excluded from the analysis. The survey contents include comprehensive information about prenatal care history, dietary behaviors, parental health status, family socioeconomic status, and the growth and development of the offspring. A detailed history of the TBCS has been previously provided elsewhere [1,24,25].

Since 2013, researchers could apply the TBCS database for clinical investigation under the permission and supervision of the Taiwan Ministry of Health and Welfare. Our data analysis was conducted at the Health and Welfare Data Science Center, between December 2019 and June 2020. This study was approved by the Institute Review Board of National Taiwan University Hospital Hsin-Chu Branch (IRB No. 107-021-E).

### 2.2. Maternal Pre-pregnancy BMI Evaluation

Maternal pBMI was obtained and calculated from the self-reported pre-pregnancy weight and height of mothers. The pre-pregnancy body weight and height of study participants were obtained from the self-reported responses of the questionnaire surveyed by face-to-face interviews, at 6 months postpartum. All women were categorized into four subgroups according to the classification of WHO: underweight (BMI < 18.5 kg/m^2^), normal weight (BMI 18.5~25 kg/m^2^), overweight (BMI 25~30 kg/m^2^) and obese (BMI ≥ 30 kg/m^2^) [26].

### 2.3. Breastfeeding Outcome Definition

The main outcome in this study is the duration of any breastfeeding, which was determined from participants’ response to the following question: “When did you completely stop feeding him (her) breastmilk?” This question was used to assess the duration of breastfeeding in the survey, at 18 months postpartum. We categorized the duration of breastfeeding into “never breastfed”, “any breastfeeding for 2 months”, “any breastfeeding for 4 months”, and “any breastfeeding for 6 months” for further analysis. In addition, we also categorized the breastfeeding duration as 0, 0–2, 2–4, 4–6, and 6 months or more, for ordinal logistic regression model analysis. Because there were no complete food or dietary records of any liquids or water other than breastmilk, for the restrictive definition of exclusive breastfeeding [27], we were not able to determine exclusive breastfeeding duration in our cohort. Consequently, we reported our outcome as any breastfeeding duration. Gestational weight gain (GWG) was defined as the difference in self-reported predelivery weight and pre-pregnancy weight. GWG was further categorized into three subgroups according to the Institute of Medicine (IOM) recommendation during pregnancy as inadequate, within the normal range or excessive GWG (recommended GWG: underweight: 12.5~18 kg; normal weight: 11.5~16 kg; overweight: 7~11.5 kg; obesity: 5~9 kg) [28].

### 2.4. Other Covariates

Potential confounders of the association between pBMI and breastfeeding were obtained from the surveys at 6 and 18 months postpartum, including maternal age at childbearing, maternal educational status, parity, smoking during pregnancy, gestational diabetes, delivery mode, employment status at 6 months postpartum, preterm birth, maternal nationality, urbanicity of living area, gestational weight gain, dyad separation, and marital status. Maternal childbearing age was categorized as “<25”, “25–34” and “≥35”. Maternal educational status was categorized as “below college” and “college or above”. Parity was categorized as “primiparous” and “multiparous”. Information about preterm birth was obtained and confirmed by birth certificate data. Immigrant mothers were defined by the nationality reported on the survey question at 6 months postpartum. The urbanicity of the living area was categorized as “urban”, “town”, and “rural area”. Dyad separation was defined if the offspring was admitted to hospital after birth for any reasons. Employment status at 6 months postpartum (returned to work) was defined if the mothers returned to work before 6 months postpartum.

### 2.5. Statistical Analysis

All analyses were conducted by using SAS statistical software version 9.4 (SAS Institute, Cary, NC, USA). Continuous and categorical variables were compared through the analysis of variance (ANOVA) and chi-square tests. The outcome of interest, namely, the categories of breastfeeding duration associated with pBMI, was evaluated by two stages of analysis. In the first stage, crude and multivariable ordinal logistic regression models were used, considering the ordinal outcomes of breastfeeding duration as 0, 0–2, 2–4, 4–6, and 6 months or more. This method could provide estimates of the effect of maternal BMI status on the likelihood of longer or shorter breastfeeding duration. We defined the shorter duration of breastfeeding as higher order in the outcome. An odds ratio of more than 1 may indicate a higher likelihood of shorter breastfeeding duration. Then, in the second stage, we performed a multivariable logistic regression model adjusted for maternal age, maternal educational status, parity, gestational diabetes, delivery mode, smoking during pregnancy, employment status at 6 months postpartum, preterm birth, maternal nationality, urbanicity, dyad separation, and marital status. For the risk adjustment and proper analyses, multicollinearity was assessed by using variance inflation factor (VIF) [29]. The VIFs values exceeding 10 were considered as signs of serious multicollinearity which may require correction. In our study, there was no serious multicollinearity presented in all the factors assessed in the regression models. The risk of any breastfeeding cessation at 2, 4, and 6 months postpartum is reported as an odds ratio (OR), with a 95% confidence interval (95% CI). In the ordinal regression model, the risk of shorter breastfeeding duration is reported as an OR with 95% Wald confidence limits. Normal-weight women were the reference group. To explore the interaction between pBMI and GWG on breastfeeding duration, we further divided our study population into 12 subgroups by different pBMI and GWG categories, and compared the risk among them. Normal-weight women with GWG within the IOM recommendation were the reference group. Considering that parity might be another important contributor to maternal obesity [16,23], we further examined the association by subgroup analysis stratified by parity.

## 3. Results

### 3.1. Clinical Characteristics of the Study Participants

There were 21,248 women who completed the survey at 6 months postpartum initially. Participants with missing or invalid data, maternal major disease or pre-existing diabetes, birth defects, or multiple pregnancies were excluded, and 18,312 women (86.2% of the original sample) were enrolled in this study (Figure S1). The clinical characteristics of the study participants are summarized in Table 1. The prevalence of underweight, normal weight, overweight and obesity was 20.1%, 69.8%, 8.2%, and 1.9%, respectively. Underweight women were significantly younger than normal-weight women, and most of them were primiparous.

The mean breastfeeding duration was longer in normal-weight mothers than in mothers in other pBMI categories. The average breastfeeding duration in our study participants was 4.17 months, and more normal-weight mothers continued breastfeeding postpartum (Table 2).

### 3.2. Association between pBMI and Breastfeeding Duration

Table 3 showed the association of maternal pBMI status and breastfeeding duration in the crude and adjusted ordinal logistic regression model. After adjustments, underweight and obese women had a greater odds ratio of shorter breastfeeding duration compared with normal-weight women.

The associations between pBMI and any breastfeeding duration are listed in Table 4. Women with normal pre-pregnancy weight status were the reference group. Maternal pre-pregnancy obesity was a risk factor for the early cessation of any breastfeeding at 2 months postpartum (aOR = 1.36, 95% CI = 1.09–1.7). In contrast, underweight women still had a greater odds ratio of early breastfeeding cessation at 2, 4, and 6 months postpartum than did normal-weight women in the adjusted model. Table S1 summarizes the association between breastfeeding duration and GWG, based on the pre-pregnancy BMI, and women with GWG within the IOM recommendation were the reference group. After adjustments were made for confounders, the odds ratio of any breastfeeding cessation at 2 and 4 months postpartum was higher in women with inadequate GWG. Women with excessive GWG had a higher odds ratio of early breastfeeding cessation at 2 months postpartum. Another logistic regression model analysis was done after excluding women who never breastfed (Table S2), and the findings showed similar results, as in the original analysis model.

To explore the interaction between pBMI and GWG on the duration of any breastfeeding, study participants were divided into 12 subgroups according to pBMI and GWG categories. Women with normal weight and GWG within the IOM recommendation were defined as the reference group. In the multivariable logistic regression model adjusted for confounders, underweight women with all GWG categories had a consistently greater odds ratio of failure to continue breastfeeding at 2, 4, and 6 months postpartum (Table 5). Normal-weight women with excessive GWG and obese women with inadequate GWG also had an increased odds ratio of early breastfeeding cessation at 2 and 4 months postpartum, while the odds ratio was attenuated to nonsignificant at 6 months.

### 3.3. Subgroup Analysis by Parity

Because the prevalence of maternal obesity may be higher in multiparous women, to assess the effect of pBMI on breastfeeding associated with parity, we further analyzed the association between pBMI and breastfeeding, stratified by primiparous and multiparous women. For women of pre-pregnancy underweight status, primiparous mothers had an increased OR of any breastfeeding cessation at 2, 4, and 6 months postpartum. Maternal obesity was only associated with a significant higher risk of breastfeeding cessation at 2 months postpartum among primiparous mothers (Table 6).

## 4. Discussion

In the present study, our findings indicated that maternal obesity was associated with a shorter duration of breastfeeding compared with normal weight, and obese mothers also had a greater risk of breastfeeding cessation at 2 months postpartum. In the ordinal logistic regression model, maternal underweight and obesity were associated with a shorter duration of breastfeeding. Moreover, underweight mothers were less likely to sustain breastfeeding, and the risks of early breastfeeding cessation postpartum at different time points were consistent in the adjusted logistic regression model and subgroup analysis stratified by parity. Our results using data from a nationally representative cohort in Taiwan indicated that maternal underweight and obesity may be important risk factors for breastfeeding practices.

Our study findings are consistent with those of previous studies, demonstrating that maternal obesity was inversely associated with breastfeeding duration [16,19,20,23,30]. Obese women were at greater risk of an inability to sustain any breastfeeding at 2 months postpartum. In our cohort, the obese rate is lower in primiparous mothers. However, the adjusted odds ratios for obese women to stop breastfeeding were not the same, and the aOR was greater in primiparous women. A potential explanation could be contributed to more breastfeeding experiences in multiparous mothers. In contrast to the effect of obesity, underweight women had an extensively greater risk of failure to continue breastfeeding at 2, 4, and 6 months postpartum.

Breastfeeding behavior is complex. It is well established that maternal obesity may contribute to many adverse perinatal outcomes, including preterm birth [31] and cesarean delivery [32], which are common risk factors for unsuccessful breastfeeding. Obese women may have other negative characteristics, such as lower socioeconomic status or educational level, that make it challenging for them to continue breastfeeding [33].

Less is known about the association between underweight and breastfeeding. In contrast to most of the previous studies on obesity and breastfeeding in Western countries [16,19,20,30], our findings indicated that maternal pre-pregnancy underweight also has a negative effect on the duration of breastfeeding. In a systematic review and meta-analysis study, Huang et al. [10] reported that maternal pre-pregnancy underweight status was associated with the initiation of breastfeeding only in sensitivity analysis involving studies with sample sizes of less than 500. In addition, only one Chinese study was enrolled in the meta-analysis study for investigation [17]. Since the prevalence of underweight is low in most Western countries [11], the effect of underweight on breastfeeding duration may need more studies to clarify the possible influences of pBMI from different countries and populations.

The potential explanations for the inconsistent results in previous literature may include differences in study designs, definitions of breastfeeding, definitions of pre-pregnancy BMI, adjusted confounding factors, race/ethnicity and sample sizes. Zhu et al. [21] reported that underweight before pregnancy increased the risk of termination of any breastfeeding within 2 months postpartum, while another study by Giovannini et al. [22] showed no association between underweight and breastfeeding duration. It is important to note that these two studies defined underweight mothers according to different criteria (Zhu: <18.44 kg/m^2^; Giovannini: <19.8 kg/m^2^). In another study, Guelinckx et al. [20] found that underweight in pre-pregnancy was associated with low intentions and low initiation of breastfeeding, and they defined underweight status according to the WHO classification (<18.5 kg/m^2^), which is the same definition we used. However, they only enrolled 50 women in each category, and could not assess the risk by a comprehensive adjusted model, due to the small sample size. An insufficient number of participants may limit the ability of the evidence to unveil the effect of underweight on breastfeeding practices, and to fully investigate this issue at the population level. Currently, the evidence of the association between maternal pre-pregnancy underweight and breastfeeding duration is still inconclusive. Researchers in the abovementioned studies all applied the same measurement as self-reported body weight and measured body height for pBMI calculation [20,21,22]. Moreover, women tend to underestimate their body weight before pregnancy [34], which may bias the effect of pre-pregnancy underweight on the duration of breastfeeding. In the future, consistent and reliable definitions of pBMI and criteria for BMI statuses should be emphasized, to compare the differences between each study accurately.

The potential mechanisms for lower breastfeeding rate in obese women might be due to decreased prolactin secretion response to suckling [14] and delayed lactogenesis II [15]. However, the underlying mechanism behind underweight and breastfeeding behavior remains unknown. The factors contributing to breastfeeding behaviors are multifaceted, and possible reasons could be considered for the low breastfeeding rate among underweight women. Underweight women may have more unfavorable background characteristics to continue breastfeeding [9]. In our study, 62.4% of underweight women were primiparous; multiparous women may have more experience with breastfeeding, and parity may be another important contributor to breastfeeding. However, in our subgroup analysis by parity, the effect of underweight persisted, which means that there might be more unmeasured risk factors for evaluation. Notably, regarding the possibility of a higher prevalence of low-birth-weight (LBW) infants among underweight women in the TBCS cohort [24], these vulnerable babies are also at an increased risk of dyad separation after birth and early breastfeeding cessation, and these interactions among SGA, LBW and underweight mothers may contribute to early breastfeeding termination [9,35].

### Strengths and Limitations

To our knowledge, the present study is the first to comprehensively investigate the association between pBMI and breastfeeding duration in a large-scale population cohort in an Asian population. Furthermore, in contrast to previous literature, the main strength of our study is its focus on differences in the effects of underweight on breastfeeding behaviors. The richness of information also allows us to analyze the contribution of pBMI in the multivariable adjusted model, stratified by primiparous and multiparous mothers.

Our study has several limitations. First, information on pBMI and breastfeeding duration was obtained from self-reported data. Although previous studies have indicated that maternal recall for pre-pregnancy weight, height and GWG were reliable [36,37] and that maternal recall for breastfeeding duration was also reliable after 3 years postpartum [38], recall bias may still exist, and the misclassification of outcomes should be considered. Given that bias of outcome misclassification is usually toward the null, our findings may underestimate the impact of pBMI on breastfeeding behaviors [39]. Second, we could only provide information on any breastfeeding duration, and not on exclusive breastfeeding, which is recommended by the WHO [4] and the AAP [5]. Given the restrictive definition of exclusive breastfeeding [27], we could not determine from the information in the questionnaires whether infants consumed any liquids or food in addition to breastmilk or formula. We can only define the outcome as any breastfeeding duration in our analysis. Third, prenatal data for maternal breastfeeding plans, detailed dietary behaviors, employment status before 6 months postpartum, treatment and diagnosis for dyad separation [6], paternal support [7], maternal perception of lacking sufficient milk supply, mastitis, infants’ failure to thrive [8], predelivery breastfeeding education [9] and psychological factors may influence the initiation and duration of breastfeeding. These factors might contribute to the causality between pBMI and breastfeeding duration. Given that our study design was an observational study, causality for the association could not be discussed in detail. Additional studies to explore the causal mechanisms between them are warranted. Fourth, our data were collected from 2006 to 2007; therefore, our results might not be fully generalized to current clinical practices.

Considering that improvements in breastfeeding rates may benefit both mothers and children, and that current breastfeeding rates are still not sufficient worldwide, our research has important public health implications to encourage healthy weight before conception, especially for underweight women of reproductive age, which may help them maintain breastfeeding for a longer period of time postpartum. Healthcare providers should raise concerns about the threat of low breastfeeding rates, regarding the long-term impact on maternal and child health. Maternal undernutrition and obesity remain significant global health issues in women of reproductive age. Nutritional status before conception has major implications for the long-term health outcomes for mothers and children [40]. Evidence-based maternity care practices and policies could help to improve breastfeeding [41]; however, underweight and obese mothers might need additional and special assistance to increase their willingness to initiate breastfeeding and their success of continuing breastfeeding for longer periods. Optimizing maternal BMI during the pre-conception period is essential, and nutrition-specific interventions should be considered, to improve maternal and child health outcomes in many areas.

## 5. Conclusions

Our study findings indicate that maternal underweight and obesity may have a negative impact on breastfeeding duration. Healthcare providers should continue to improve breastfeeding support, especially for women who may have a higher likelihood of early breastfeeding cessation. Given the inconsistent findings in previous literature, additional studies are needed to better understand the impact of maternal underweight on breastfeeding, and the mechanism behind this association. Interventions to improve breastfeeding rates and provide education regarding a healthy weight before conception are necessary for the promotion of maternal and child health.

## Figures and Tables

**Table 1:** Clinical characteristics of the study participants.

	Underweight	Normal	Overweight	Obesity	All	*p*-Value *
N	3686	12,787	1498	341	18,312	
Maternal and gestational factors						
Pre-pregnancy BMI	17.5 (0.8)	20.9 (1.7)	26.9 (1.3)	32.8 (2.6)	21 (3.2)	
Maternal age	26.8 (4.7)	28.6 (4.8)	29.5 (4.9)	28.9 (4.8)	28.3 (4.9)	<0.0001
Parity						<0.0001
Primiparous	2301 (62.4)	6420 (50.2)	537 (35.9)	123 (36.1)	9381 (51.2)	
Smoking during pregnancy	176 (4.8)	367 (2.9)	57 (3.8)	14 (4.1)	614 (3.4)	<0.0001
Gestational diabetes	39 (1.1)	236 (1.9)	78 (5.2)	25 (7.3)	378 (2.1)	<0.0001
Cesarean delivery	980 (26.6)	4077 (31.9)	661 (44.1)	198 (58.1)	5916 (32.3)	<0.0001
Preterm delivery	224 (6.1)	774 (6.1)	140 (9.4)	44 (12.9)	1182 (6.5)	<0.0001
Dyad separation	85 (2.3)	267 (2.1)	50 (3.3)	17 (5)	419 (2.3)	0.0001
Gestational weight gain						<0.0001
Inadequate	1410 (38.3)	3599 (28.2)	170 (11.4)	37 (10.9)	5216 (28.5)	
Within normal range	1606 (43.6)	5419 (42.4)	548 (36.6)	99 (29)	7672 (41.9)	
Excessive	670 (18.2)	3769 (29.5)	780 (52.1)	205 (60.1)	5424 (29.6)	
Infant sex, male	1855 (50.3)	6696 (52.4)	761 (50.8)	182 (53.4)	9494 (51.9)	0.12
Socioeconomic factors						
Maternal education						<0.0001
College or above	596 (16.2)	2885 (22.6)	222 (14.8)	31 (9.1)	3734 (20.4)	
Urbanicity of living area						<0.01
Rural	929 (25.2)	3144 (24.6)	409 (27.3)	99 (29)	4581 (25)	
Town	10,000 (27.1)	3532 (27.6)	442 (29.5)	100 (29.3)	5074 (27.7)	
Urban	1757 (47.7)	6111 (47.8)	647 (43.2)	142 (41.6)	8657 (47.3)	
Immigrant mothers	688 (18.7)	1638 (12.8)	63 (4.2)	6 (1.8)	2395 (13.1)	<0.0001
Marital status (yes)	3560 (96.6)	12510 (97.8)	1463 (97.7)	323 (94.7)	17,856 (97.5)	<0.0001
Employment status at 6 months postpartum (returned to work)	1986 (53.9)	7709 (60.3)	862 (57.5)	182 (53.4)	10,739 (58.6)	<0.0001

Data are presented as mean (SD), numbers (%) or medians. * By chi-square test or ANOVA.

**Table 2:** Breastfeeding rates and duration among women in different BMI categories.

Breastfeeding Duration	Underweight	Normal	Overweight	Obesity	All	*p*-Value *
Total duration (months)	3.75 ± 4.86	4.33 ± 5.25	4.16 ± 5.38	3.16 ± 4.61	4.17 ± 5.18	<0.0001
Never breastfed	614 (16.7)	1930 (15.1)	300 (20)	91 (26.7)	2935 (16)	<0.0001
0–2 months	1090 (29.6)	3496 (27.3)	405 (27)	100 (29.3)	5091 (27.8)	
2–4 months	925 (25.1)	2905 (22.7)	311 (20.8)	64 (18.8)	4205 (23)	
4–6 months	256 (6.9)	1038 (8.1)	93 (6.2)	20 (5.9)	1407 (7.7)	
6 months or more	801 (21.7)	3418 (26.8)	389 (26)	66 (19.3)	4674 (25.5)	

Data are presented as numbers (%) or means ± standard deviations. * By chi-square test or ANOVA.

**Table 3:** Association between pre-pregnancy body mass index status and breastfeeding duration by ordinal logistic regression model.

	Crude OR	95% Wald Confidence Limits	Adjusted OR *	95% Wald Confidence Limits
Underweight	1.22	1.15, 1.31	1.21	1.13, 1.29
Normal	Reference	-	Reference	-
Overweight	1.21	1.09, 1.33	0.98	0.89, 1.08
Obese	1.83	1.5, 2.23	1.25	1.02, 1.53

* Adjusted for maternal age, parity, maternal education, maternal immigration status, urbanicity of living area, maternal gestational diabetes, cesarean delivery, employment status at 6 months postpartum, preterm delivery, marital status, dyad separation, gestational weight gain and smoking during pregnancy.

**Table 4:** Association between pre-pregnancy body mass index status and early breastfeeding cessation at different periods.

	Crude OR	95% CI	Adjusted OR *	95% CI
Breastfeeding cessation at 2 months postpartum				
Underweight	1.17	1.09, 1.26	1.11	1.03, 1.2
Normal	Reference	-	Reference	-
Overweight	1.21	1.08, 1.34	1.05	0.94, 1.17
Obese	1.73	1.39, 2.15	1.36	1.09, 1.7
Breastfeeding cessation at 4 months postpartum				
Underweight	1.33	1.23, 1.44	1.32	1.21, 1.43
Normal	Reference	-	Reference	-
Overweight	1.13	1.01, 1.26	0.96	0.85, 1.08
Obese	1.59	1.24, 2.03	1.21	0.94, 1.57
Breastfeeding cessation at 6 months postpartum				
Underweight	1.31	1.2, 1.43	1.3	1.18, 1.42
Normal	Reference	-	Reference	-
Overweight	1.04	0.92, 1.18	0.87	0.77, 1
Obese	1.52	1.16, 1.99	1.15	0.87, 1.52

* Adjusted for maternal age, parity, maternal education, maternal immigration status, urbanicity of living area, maternal gestational diabetes, cesarean delivery, employment status at 6 months postpartum, preterm delivery, marital status, dyad separation, gestational weight gain and smoking during pregnancy.

**Table 5:** Association of pre-pregnancy body mass index, gestational weight gain and early breastfeeding cessation at different periods.

pBMI and GWG Categories	2 Months Postpartum		4 Months Postpartum		6 Months Postpartum	
	aOR *	95% CI	aOR *	95% CI	aOR *	95% CI
Normal weight						
With adequate GWG	Ref	-	Ref	-	Ref	-
Inadequate GWG	1.12	1.02, 1.22	1.09	0.99, 1.2	1	0.9, 1.1
Excessive GWG	1.12	1.03, 1.22	1.12	1.02, 1.23	1.08	0.98, 1.2
Underweight						
With adequate GWG	1.17	1.04, 1.32	1.33	1.17, 1.51	1.29	1.13, 1.49
Inadequate GWG	1.18	1.05, 1.34	1.44	1.26, 1.65	1.3	1.12, 1.5
Excessive GWG	1.23	1.04, 1.46	1.45	1.2, 1.76	1.42	1.15, 1.76
Overweight						
With adequate GWG	1.09	0.91, 1.31	1.02	0.84, 1.24	0.96	0.78, 1.18
Inadequate GWG	0.99	0.72, 1.36	1	0.71, 1.41	0.9	0.63, 1.3
Excessive GWG	1.18	1.01, 1.38	1.03	0.87, 1.21	0.87	0.73, 1.04
Obese						
With adequate GWG	1.44	0.95, 2.16	1.46	0.9, 2.35	1.29	0.77, 2.17
Inadequate GWG	5.38	2.32, 12.51	2.82	1.07, 7.42	3.21	0.96, 10.7
Excessive GWG	1.22	0.9, 1.62	1.11	0.8, 1.52	1.02	0.72, 1.45

* Adjusted for maternal age, parity, maternal education, maternal immigration status, urbanicity of living area, maternal gestational diabetes, cesarean delivery, employment status at 6 months postpartum, preterm delivery, marital status, dyad separation, and smoking during pregnancy. Abbreviations: pBMI, pre-pregnancy body mass index; GWG, gestational weight gain; aOR, adjusted odds ratio.

**Table 6:** Subgroup analysis for the risk of breastfeeding cessation at different postpartum periods stratified by parity.

Subgroup	Primiparous Women		Multiparous Women	
	aOR *	95% CI	aOR *	95% CI
Breastfeeding cessation at 2 months postpartum				
Underweight	1.11	1.01, 1.23	1.12	0.99, 1.26
Normal	Ref	-	Ref	-
Overweight	1.2	0.99, 1.43	0.97	0.84, 1.12
Obese	1.56	1.06, 2.26	1.25	0.94, 1.66
Breastfeeding cessation at 4 months postpartum				
Underweight	1.34	1.2, 1.49	1.29	1.13, 1.47
Normal	Ref	-	Ref	-
Overweight	1.04	0.85, 1.27	0.91	0.78, 1.06
Obese	1.36	0.88, 2.1	1.15	0.84, 1.59
Breastfeeding cessation at 6 months postpartum				
Underweight	1.35	1.19, 1.52	1.22	1.06, 1.41
Normal	Ref	-	Ref	-
Overweight	0.95	0.76, 1.18	0.83	0.7, 0.97
Obese	1.5	0.91, 2.49	1	0.71, 1.41

* Adjusted for maternal age, maternal education, maternal immigration status, urbanicity of living area, maternal gestational diabetes, cesarean delivery, employment status at 6 months postpartum, preterm delivery, marital status, dyad separation, gestational weight gain, and smoking during pregnancy. Abbreviations: aOR, adjusted odds ratio.

## Supplementary Materials

The following are available online at https://www.mdpi.com/2072-6643/12/8/2361/s1. Figure S1. Flow chart of the study population. Table S1. Breastfeeding rates and duration among women in different gestational weight gain categories. Table S2. Association between pre-pregnancy body mass index status and early breastfeeding cessation at different periods after excluding mothers who never breastfed.

## References

[1] Chiu W.C., Liao H.F., Chang P.J., Chen P.C., Chen Y.C. (2011). Duration of breast feeding and risk of developmental delay in Taiwanese children: A nationwide birth cohort study. Paediatr. Perinat. Epidemiol..

[2] Victora C.G., Bahl R., Barros A.J., Franca G.V., Horton S., Krasevec J., Murch S., Sankar M.J., Walker N., Rollins N.C. (2016). Breastfeeding in the 21st century: Epidemiology, mechanisms, and lifelong effect. Lancet.

[3] Binns C., Lee M., Low W.Y. (2016). The long-term public health benefits of breastfeeding. Asia Pac. J. Public Health.

[4] World Health Organization (2003). Global Strategy for Infant and Young Child Feeding.

[5] (2012). Section on Breastfeeding. Breastfeeding and the use of human milk. Pediatrics.

[6] Chang P.C., Li S.F., Yang H.Y., Wang L.C., Weng C.Y., Chen K.F., Chen W., Fan S.Y. (2019). Factors associated with cessation of exclusive breastfeeding at 1 and 2 months postpartum in Taiwan. Int. Breastfeed. J..

[7] Abbass-Dick J., Brown H.K., Jackson K.T., Rempel L., Dennis C.L. (2019). Perinatal breastfeeding interventions including fathers/partners: A systematic review of the literature. Midwifery.

[8] Gianni M.L., Bettinelli M.E., Manfra P., Sorrentino G., Bezze E., Plevani L., Cavallaro G., Raffaeli G., Crippa B.L., Colombo L. (2019). Breastfeeding Difficulties and Risk for Early Breastfeeding Cessation. Nutrients.

[9] Cohen S.S., Alexander D.D., Krebs N.F., Young B.E., Cabana M.D., Erdmann P., Hays N.P., Bezold C.P., Levin-Sparenberg E., Turini M. (2018). Factors associated with breastfeeding initiation and continuation: A meta-analysis. J. Pediatr..

[10] Huang Y., Ouyang Y.Q., Redding S.R. (2019). Maternal prepregnancy body mass index, gestational weight gain, and cessation of breastfeeding: A systematic review and meta-analysis. Breastfeed. Med..

[11] Poston L., Caleyachetty R., Cnattingius S., Corvalan C., Uauy R., Herring S., Gillman M.W. (2016). Preconceptional and maternal obesity: Epidemiology and health consequences. Lancet Diabetes Endocrinol..

[12] Ogden C.L., Carroll M.D., Fryar C.D., Flegal K.M. (2015). Prevalence of obesity among adults and youth: United States, 2011–2014. NCHS Data Brief.

[13] Catalano P.M., Shankar K. (2017). Obesity and pregnancy: Mechanisms of short term and long term adverse consequences for mother and child. BMJ.

[14] Rasmussen K.M., Kjolhede C.L. (2004). Prepregnant overweight and obesity diminish the prolactin response to suckling in the first week postpartum. Pediatrics.

[15] Preusting I., Brumley J., Odibo L., Spatz D.L., Louis J.M. (2017). Obesity as a predictor of delayed lactogenesis II. J. Hum. Lact..

[16] Boudet-Berquier J., Salanave B., Desenclos J.C., Castetbon K. (2018). Association between maternal prepregnancy obesity and breastfeeding duration: Data from a nationwide prospective birth cohort. Matern. Child Nutr..

[17] Tao X.Y., Huang K., Yan S.Q., Zuo A.Z., Tao R.W., Cao H., Gu C.L., Tao F.B. (2017). Pre-pregnancy BMI, gestational weight gain and breast-feeding: A cohort study in China. Public Health Nutr..

[18] Masho S.W., Cha S., Morris M.R. (2015). Prepregnancy obesity and breastfeeding noninitiation in the United States: An examination of racial and ethnic differences. Breastfeed. Med..

[19] Winkvist A., Brantsaeter A.L., Brandhagen M., Haugen M., Meltzer H.M., Lissner L. (2015). Maternal Prepregnant Body Mass Index and Gestational Weight Gain Are Associated with Initiation and Duration of Breastfeeding among Norwegian Mothers. J. Nutr..

[20] Guelinckx I., Devlieger R., Bogaerts A., Pauwels S., Vansant G. (2012). The effect of pre-pregnancy BMI on intention, initiation and duration of breast-feeding. Public Health Nutr..

[21] Zhu P., Hao J., Jiang X., Huang K., Tao F. (2013). New insight into onset of lactation: Mediating the negative effect of multiple perinatal biopsychosocial stress on breastfeeding duration. Breastfeed. Med..

[22] Giovannini M., Radaelli G., Banderali G., Riva E. (2007). Low prepregnant body mass index and breastfeeding practices. J. Hum. Lact..

[23] Nomura K., Kido M., Tanabe A., Ando K. (2019). Prepregnancy obesity as a risk factor for exclusive breastfeeding initiation in Japanese women. Nutrition.

[24] Chen C.N., Chen H.S., Hsu H.C. (2020). Maternal prepregnancy body mass index, gestational weight gain, and risk of adverse perinatal outcomes in Taiwan: A population-based Birth cohort study. Int. J. Environ. Res. Public Health.

[25] Hsieh C.Y., Su C.C., Shao S.C., Sung S.F., Lin S.J., Yang Y.H.K., Lai E.C. (2019). Taiwan’s national health insurance research database: Past and future. Clin. Epidemiol..

[26] World Health Organization (2000). Obesity: Preventing and Managing the Global Epidemic.

[27] Labbok M., Krasovec K. (1990). Toward consistency in breastfeeding definitions. Stud. Fam. Plan..

[28] Rasmussen K.M., Yaktine A.L., Institute of Medicine National Research Council Committee to Reexamine I O M Pregnancy Weight Guidelines (2009). The national academies collection: Reports funded by national institutes of health. Weight Gain During Pregnancy: Reexamining the Guidelines.

[29] Iezzoni L.I. (1997). Risk Adjustment for Measuring Health Care Outcomes.

[30] Baker J.L., Michaelsen K.F., Sorensen T.I., Rasmussen K.M. (2007). High prepregnant body mass index is associated with early termination of full and any breastfeeding in Danish women. Am. J. Clin. Nutr..

[31] Briere C.E., McGrath J., Cong X., Cusson R. (2014). An integrative review of factors that influence breastfeeding duration for premature infants after NICU hospitalization. J. Obstet. Gynecol. Neonatal. Nurs..

[32] Regan J., Thompson A., DeFranco E. (2013). The influence of mode of delivery on breastfeeding initiation in women with a prior cesarean delivery: A population-based study. Breastfeed. Med..

[33] Kitsantas P., Gaffney K.F., Kornides M.L. (2011). Prepregnancy body mass index, socioeconomic status, race/ethnicity and breastfeeding practices. J. Perinat. Med..

[34] Headen I., Cohen A.K., Mujahid M., Abrams B. (2017). The accuracy of self-reported pregnancy-related weight: A systematic review. Obes. Rev..

[35] Hwang W.J., Chung W.J., Kang D.R., Suh M.H. (2006). Factors affecting breastfeeding rate and duration. J. Prev. Med. Public Health.

[36] Tomeo C.A., Rich-Edwards J.W., Michels K.B., Berkey C.S., Hunter D.J., Frazier A.L., Willett W.C., Buka S.L. (1999). Reproducibility and validity of maternal recall of pregnancy-related events. Epidemiology.

[37] Hinkle S.N., Sharma A.J., Schieve L.A., Ramakrishnan U., Swan D.W., Stein A.D. (2013). Reliability of gestational weight gain reported postpartum: A comparison to the birth certificate. Matern. Child Health J..

[38] Li R., Scanlon K.S., Serdula M.K. (2005). The validity and reliability of maternal recall of breastfeeding practice. Nutr. Rev..

[39] Delgado-Rodríguez M., Llorca J. (2004). Bias. J. Epidemiol. Community Health.

[40] Fleming T.P., Watkins A.J., Velazquez M.A., Mathers J.C., Prentice A.M., Stephenson J., Barker M., Saffery R., Yajnik C.S., Eckert J.J. (2018). Origins of lifetime health around the time of conception: Causes and consequences. Lancet.

[41] Nelson J.M., Perrine C.G., Freedman D.S., Williams L., Morrow B., Smith R.A., Dee D.L. (2018). Infant feeding-related maternity care practices and maternal report of breastfeeding outcomes. Birth.

