# Supplementary material for: Association between Maternal Pre-pregnancy Body Mass Index and Breastfeeding Duration in Taiwan: A Population-Based Cohort Study
Source: Nutrients. 2020 Aug 7;12(8):2361. doi: 10.3390/nu12082361 (PMC7468738; doi:10.3390/nu12082361)
Supplement: Supplementary file 1 [file nutrients-12-02361-s001.pdf]

Supplementary material

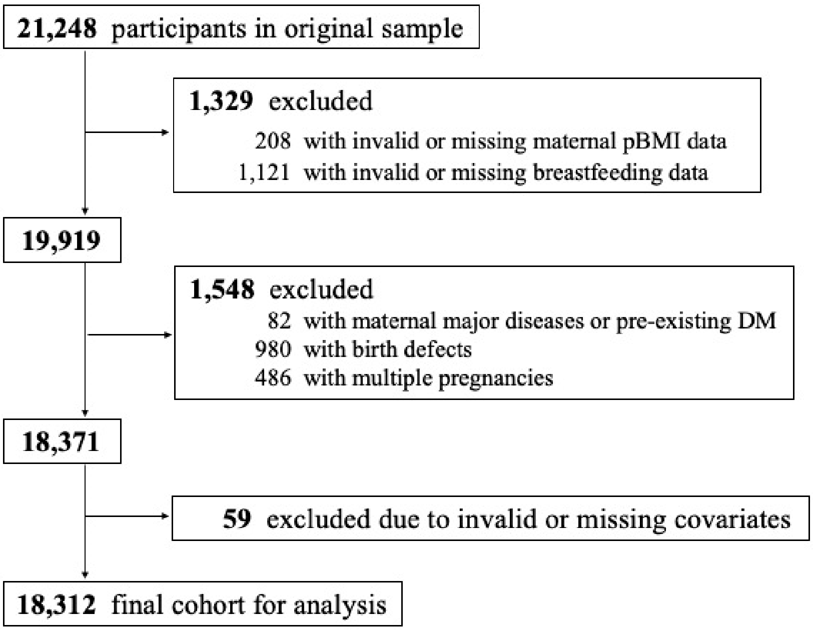

Figure S1. Flow chart of the study population.

Table S1. Breastfeeding rates and duration among women in different gestational weight gain categories.

	Crude OR	95% CI	Adjusted OR*	95% CI
Breastfeeding cessation at 2 months postpartum				
Inadequate GWG	1.11	1.03, 1.19	1.09	1.02, 1.18
Adequate GWG	Reference	-	Reference	-
Excessive GWG	1.21	1.12, 1.29	1.1	1.02, 1.18
Breastfeeding cessation at 4 months postpartum				
Inadequate GWG	1.09	1.01, 1.18	1.11	1.02, 1.2
Adequate GWG	Reference	-	Reference	-
Excessive GWG	1.18	1.01, 1.27	1.07	0.99, 1.16
Breastfeeding cessation at 6 months postpartum				
Inadequate GWG	1.01	0.93, 1.09	1.02	0.93, 1.11
Adequate GWG	Reference	-	Reference	-
Excessive GWG	1.14	1.05, 1.23	1.03	0.94, 1.12

*Adjusted for maternal age, maternal education, maternal immigration status, urbanicity of living area, maternal gestational diabetes, cesarean delivery, employment status at 6 months postpartum, preterm delivery, marital status, dyad separation, and smoking during pregnancy. Abbreviations: GWG, gestational weight gain.

Table S2. Association between pre-pregnancy body mass index status and early breastfeeding cessation at different periods after excluding mothers who never breastfed.

	**Crude OR**	**95% CI**	**Adjusted OR***	**95% CI**
Breastfeeding cessation at 2 months postpartum				
Underweight	1.16	1.07, 1.27	1.13	1.03, 1.23
Normal	Reference	-	Reference	-
Overweight	1.07	0.94, 1.21	0.9	0.79, 1.03
Obese	1.43	1.1, 1.84	1.04	0.8, 1.36
Breastfeeding cessation at 4 months postpartum				
Underweight	1.33	1.22, 1.44	1.35	1.24, 1.48
Normal	Reference	-	Reference	-
Overweight	1.03	0.92, 1.17	0.87	0.76, 0.99
Obese	1.33	1.02, 1.73	0.97	0.73, 1.27
Breastfeeding cessation at 6 months postpartum				
Underweight	1.3	1.19, 1.42	1.32	1.2, 1.46
Normal	Reference	-	Reference	-
Overweight	0.96	0.84, 1.09	0.79	0.69, 0.91
Obese	1.28	0.97, 1.7	0.92	0.68, 1.24

*Adjusted for maternal age, parity, maternal education, maternal immigration status, urbanicity of living area, maternal gestational diabetes, cesarean delivery, employment status at 6 months postpartum, preterm delivery, marital status, dyad separation, gestational weight gain and smoking during pregnancy
